# Ten‐year alcohol consumption typologies and trajectories of C‐reactive protein, interleukin‐6 and interleukin‐1 receptor antagonist over the following 12 years: a prospective cohort study

**DOI:** 10.1111/joim.12544

**Published:** 2016-08-03

**Authors:** S. Bell, G. Mehta, K. Moore, A. Britton

**Affiliations:** ^1^ Research Department of Epidemiology and Public HealthUniversity College LondonLondonUK; ^2^ UCL Institute of Liver and Digestive HealthRoyal Free CampusUniversity College LondonLondonUK

**Keywords:** alcohol, cytokines, epidemiology, inflammation, longitudinal

## Abstract

**Background:**

Moderate alcohol consumption is thought to confer cardiometabolic protective effects. Inflammatory pathways are hypothesized to partly underlie this association.

**Objectives:**

The aim of this study was to examine the association between typologies of alcohol consumption and markers of inflammation, and their rate of change over time.

**Methods:**

Data were collected from 8209 participants [69% men; mean age, 50 years (SD 6.1)] of the British Whitehall II study. Alcohol consumption typologies were defined using up to three measures during an approximately 10‐year period spanning from 1985 to 1994 as (i) stable nondrinkers, (ii) stable moderate drinkers (referent), (iii) stable heavy drinkers, (iv) nonstable drinkers and (v) former drinkers. C‐reactive protein (CRP), interleukin (IL)‐6 and IL‐1 receptor antagonist (IL‐1 RA) were measured up to three times in the following 12 years.

**Results:**

Stable moderate drinkers had lower levels of CRP than stable nondrinkers, stable heavy drinkers, former drinkers and nonstable drinkers, but there were no differences in the rate of change in CRP over time between groups. Stable nondrinkers had higher levels of IL‐6 as did stable heavy drinkers; rates of change in IL‐6 over time were also increased in the latter group. Stable nondrinkers also had higher levels of IL‐1 RA. These associations were robust to adjustment for confounding factors.

**Conclusion:**

Our novel investigation of 10‐year drinking typologies shows that stable moderate alcohol consumption is associated with a long‐term inflammatory marker profile that is consistent with conferring a reduced risk of developing coronary heart disease.

## Introduction

Moderate alcohol consumption is thought to confer cardiometabolic protective effects 1, 2, 3 and has also been demonstrated to be related to a lower risk of a plethora of other disorders of different aetiology compared to both no alcohol and heavy alcohol intake 4. Numerous biological mechanisms have been put forward to explain the proposed cardiometabolic protection 5, 6, with favourable changes in high‐density lipoprotein (HDL) cholesterol, fibrinogen and adiponectin supported by evidence from several small‐scale randomized controlled feeding trials 7. However, these factors are unlikely to entirely explain the protective effects observed for moderate consumption and cardiometabolic outcomes compared to abstinence (or the increased risk observed amongst heavier drinkers), and their causal role in the aetiology of cardiovascular disease (CVD) remains unclear 8, 9, 10, 11, 12, 13, 14.

Therefore, if the protective cardiometabolic effects observed are genuine, it is likely that other biological pathways are involved, one of which may be via pro‐inflammatory cytokines 15. For example, higher levels of the acute‐phase reactant C‐reactive protein (CRP) are associated with an increased risk of developing CVD 16 and a variety of other disease end‐points 17. Studies have demonstrated that higher levels of interleukin (IL)‐6 are associated with an increased risk of coronary heart disease (CHD) 18, including studies examining long‐term exposure to elevated IL‐6 19 or functional genetic variants of IL‐6 signalling 20, suggesting that the association is causal. IL‐1 is considered a master regulator of inflammation that triggers the release of a variety of inflammatory markers through activating the IL‐1 receptor. The IL‐1 receptor antagonist (IL‐1 RA) is an endogenous inhibitor of IL‐1 that prevents the activation of the IL‐1 receptor by either IL‐1α or IL‐1β 21. IL‐1 RA is associated with a diverse range of diseases including CVD, type 2 diabetes, certain cancers and joint diseases such as arthritis 21, 22, 23, 24, 25. In a recent large‐scale Mendelian randomization study, it was found that genetically elevated levels of IL‐1 RA were causally associated with an increased risk of CHD and abdominal aortic aneurysm 26.

The findings of studies investigating alcohol consumption and inflammatory markers have been mixed. There is limited and conflicting evidence from interventional studies, typically with sample sizes of <100 participants and over relatively short periods of time, of the association between alcohol consumption and inflammation 7, 27, 28, 29. In terms of observational studies, some investigators have found that moderate alcohol consumption is associated with lower levels of CRP 30, 31, 32 and IL‐6 32, 33 compared to no alcohol and heavy alcohol intake, whereas others have observed no association 33, 34. Studies of the association between alcohol intake in the general population and IL‐1 RA are scarce 33.

Furthermore, most previous studies have only used a single measure of alcohol intake at baseline to define the drinking behaviour of participants, assuming that it is static thereafter. However, individuals’ drinking habits change over time 35, 36, and this can affect their risk of developing disease 37. Therefore, not accounting for long‐term drinking profiles or changes in alcohol consumption can introduce bias 38, 39, 40. A classic example of such bias is the failure to separate former drinkers from never drinkers which is known to in some cases substantially impact findings and subsequent conclusions that are drawn 41, 42. Levels of CRP, IL‐6 and IL‐1 RA also change over time and can similarly influence disease risk 25, 43, 44.

In summary, studies utilizing repeat measures of alcohol consumption 35 or examining the influence of alcohol on the change in biomarker levels over time 45 are rare. Studies combining these two elements are nonexistent. We therefore aimed to examine the association between typologies of alcohol consumption over an approximately 10‐year period and markers of inflammation, including CRP, IL‐6 and IL‐1 RA, and their rate of change over the following 12 years.

## Materials and methods

### Study population

Data were obtained from the Whitehall II cohort of British civil servants 46. The study started during the period 1985–1988 (phase 1) and included 10 308 participants (6895 men) aged 35–55 years. All civil servants within this age range in 20 London‐based departments were invited by letter to participate, and 73% agreed. The first phase involved a clinical examination as well as a self‐administered questionnaire to collect information on demographic characteristics, health, lifestyle factors, work characteristics, social support and life events. Subsequent phases of data collection have alternated between postal questionnaire alone and postal questionnaire accompanied by a clinical examination. Data used in this investigation were from phases 1 (1985–1988), 2 (1989–1990), 3 (1991–1994), 5 (1997–1999) and 7 (2002–2004) of the study. The maximum available sample was 8209 participants for whom there was information on alcohol consumption, age and gender, and at least one measure of CRP, IL‐6 or IL‐1 RA.

### Ethical considerations and data access

The University College London Medical School committee on the Ethics of Human Research approved the Whitehall II study. Informed consent was obtained at baseline and renewed at each contact. Whitehall II study data, protocols and other metadata are available to *bona fide* researchers for research purposes (data sharing policy is available at http://www.ucl.ac.uk/whitehallII/data-sharing).

### Assessment of alcohol consumption

We used alcohol information collected at study phases 1, 2 and 3 to construct (approximately) 10‐year drinking typologies. Participants were asked to report the number of alcoholic drinks they had consumed in the previous week, providing information separately for beer/cider (in pints), wine (in glasses) and spirits (in measures). Drinks were converted into grams of ethanol using a conservative estimate of 8 g for each measure of spirits and glass of wine, and 16 g for each pint of beer 47. The sum of these converted measurements was then used to define total weekly amount of alcohol intake in grams. We then constructed categories of alcohol consumption based on UK guidelines for sensible drinking at the time: none, moderate (within guidelines of 8–168 g ethanol per week for men and 8–112 g for women) and heavy (intake above these guidelines).

Typologies of drinkers to remain in keeping with the results over study phases 1–3 were defined as follows: stable nondrinkers, stable moderate drinkers, stable heavy drinkers, nonstable drinkers (participants who moved between categories of consumption during observation) and former drinkers [nondrinkers at study phase 3 who had previously reported consuming alcohol (at any level) at earlier study phases]. Participants were permitted one missing alcohol value in the construction of the 10‐year alcohol typology variable. Our reference group for analyses was stable moderate drinkers 48.

### Assessment of inflammatory markers

Fasting serum samples were collected between 08.00 and 13.00, stored at −80°C and were not thawed or refrozen during storage. Although serum samples were from three different study phases, blood collection, processing and storage followed the same standard operating procedures.

CRP was measured with a high‐sensitivity immunonephelometric assay in a BN ProSpec nephelometer (Dade Behring, Milton Keynes, UK). IL‐6 was measured with a high‐sensitivity enzyme‐linked immunosorbent assay (ELISA) (R&D Systems, Oxford, UK). Values lower than the detection limit [0.154 mg L^−1^ for CRP (multiplied by 9524 to express the value in mmol L^−1^) and 0.08 pg mL^−1^ for IL‐6] were assigned a value equal to half the detection limit. We excluded samples with CRP concentrations suggestive of acute inflammation and related bacterial infection (>10 mg L^−1^) 49 (*n* = 242). To measure short‐term biological variation and laboratory error, a repeat sample was taken from 150 participants for CRP and 241 participants for IL‐6 at phase 3 [with a mean elapsed time between samples of 32 days (SD 10.5)]. Intra‐assay and interassay coefficients of variation were 4.7% and 8.3% for CRP and 7.5% and 8.9% for IL‐6, respectively.

Serum IL‐1 RA was measured in a diabetes case–cohort sample 25, 50 with the Quantikine ELISA kit (R&D Systems, Wiesbaden, Germany). All assays were performed consecutively in the same laboratory (German Diabetes Center), and samples from different study phases from the same participant were always measured using the same ELISA plate to minimize assay imprecision. Mean intra‐assay and interassay coefficients of variation were 2.6% and 7.9%, respectively. The limit of detection was 14 pg mL^−1^ (all samples were above the limit of detection).

### Covariates

Age, sex, ethnicity (White or non‐White), prevalent CHD (clinically verified events) and type 2 diabetes (cases defined by oral glucose tolerance tests and/or use of diabetes medication) at study phase 3 were entered into our statistical models as time‐invariant predictors.

Time‐varying covariates included in our models were socio‐economic position [defined using either current or last recorded civil service employment grade as high (unified grades 1–7), intermediate (executive officers) or low (clerical or support staff), as previously described 51] and health behaviours including smoking status (never, former and current) and physical activity (lowest sex‐specific quartile of combined hours of moderate and vigorous physical activity defined as ‘physically inactive’). Diet quality was classified as poor or good using three questions on the type of milk and bread participants usually consumed and their frequency of fruit and vegetable intake. For each dietary component, a score of one was assigned to poor diet quality indicators (whole milk, white bread, fruit and vegetable intake less than daily), and a summed score ≥2 was used to classify poor diet quality 52. Body mass index (BMI) was calculated using the standard formula, and participants were classified as normal weight (18.5–24.9 kg m^−2^), overweight/obese (≥25 kg m^−2^) or underweight (<18.5 kg m^−2^) using thresholds adopted by the World Health Organisation.

### Statistical analysis

Differences in sample characteristics by drinking typologies were assessed using chi‐square test or one‐way anova. To examine the association between alcohol typologies and trajectories of inflammatory markers, we used linear mixed models with time from phase 3 in years as the time metric (allowing for individually varying times of observation). We included random effects for the intercept and time and allowed for random effects to covary. As CRP, IL‐6 and IL‐1 RA were positively skewed, we used natural logarithm‐transformed values in all analyses (predictions were calculated using these models and then back‐transformed to their original scale for graphical presentation). Preliminary analyses revealed no evidence of effect modification between drinking typologies and sex in the rate of change in any inflammatory marker. Therefore, two models are presented for each outcome, one with adjustments only for age and sex and the other with adjustments for all covariates described above. These models include terms for the difference in inflammatory markers at study phase 3 by alcohol group (intercept differences) as well as interactions between drinking categories and time to describe differences in the rate of change in inflammatory markers per year of follow‐up by alcohol typologies. We also include analyses with alcohol intake categories defined using data from phase 3 assessment only, presented in the Online Supplementary Material, so that findings from the main analysis can be compared to those that would have been obtained using the conventional approach of only using alcohol intake assessed at one time‐point (as in the main analyses presented, these models include terms describing differences in baseline values of inflammatory markers as well as their rate of change over time). An α level of <0.05 was considered statistically significant. All analyses were conducted using Stata 14.1 StataCorp (College Station, Texas, USA).

## Results

### Descriptive statistics

The characteristics of participants at study phase 3 are presented in Table 1. The mean age of participants was 50 years (SD 6.1). Almost 70% of the participants were men, and the majority were of White ethnicity and high or intermediate socio‐economic position. Approximately half of the participants had never smoked whilst 14% were current smokers. The majority of participants were physically active and made good dietary choices. Half of the sample had a BMI considered to be in the normal range, and 47% were considered overweight. Prevalent CHD (3%) or type 2 diabetes (1%) was rare amongst participants. During follow‐up, there were 696 and 873 incident cases of CHD and type 2 diabetes, respectively. Descriptive statistics for changes in alcohol consumption and inflammatory markers are presented in Table S1.

**Table 1:** Characteristics at phase 3 of Whitehall II study by 10‐year drinking typologies

	Stable nondrinker		Stable moderate drinker		Stable heavy drinker		Nonstable drinker		Former drinker		Total		*P* for diff
	*n*	%	*n*	%	*n*	%	*n*	%	*n*	%	*n*	%	
Age, mean (SD) years	746	51.3 (6.1)	3909	50.3 (6.1)	671	48.8 (5.7)	2052	49.6 (6.0)	831	50.9 (6.2)	8209	50.1 (6.1)	<0.001
Sex													
Male	345	46	2885	74	570	85	1430	70	465	56	5695	69	
Female	401	54	1024	26	101	15	622	30	366	44	2514	31	<0.001
Ethnicity													
White	494	66	3642	93	658	98	1895	92	731	88	7420	90	
Non‐White	252	34	267	7	13	2	157	8	100	12	789	10	<0.001
SEP													
High	106	15	1719	45	339	51	761	38	166	20	3091	38	
Intermediate	310	43	1654	43	293	44	962	48	423	51	3642	45	
Low	308	43	463	12	29	4	284	14	237	29	1321	16	<0.001
Smoking status													
Never smoker	418	63	1802	50	162	26	795	41	387	50	3564	47	
Ex‐smoker	144	22	1368	38	323	51	827	43	263	34	2925	39	
Current smoker	98	15	406	11	143	23	311	16	129	17	1087	14	<0.001
Physical activity level													
Active	642	89	3649	95	629	95	1868	93	747	90	7535	94	
Inactive	81	11	183	5	31	5	137	7	81	10	513	6	<0.001
Dietary choice quality													
Good	511	71	2978	78	478	72	1532	76	612	74	6111	76	
Poor	212	29	857	22	182	28	473	24	217	26	1941	24	<0.001
BMI category													
Normal weight	342	48	2035	54	292	46	982	50	418	52	4069	52	
Overweight or obese	361	50	1691	45	347	54	964	49	382	47	3745	48	
Underweight	16	2	37	1	3	1	14	1	6	1	76	1	<0.001
Prevalent CHD at phase 3													
No	709	95	3791	97	654	98	1987	97	804	97	7945	97	
Yes	37	5	118	3	17	3	65	3	27	3	264	3	0.07
Prevalent type 2 diabetes at phase 3													
No	716	96	3868	99	660	98	2024	99	818	98	8086	99	
Yes	30	4	41	1	11	2	28	1	13	2	123	2	<0.001

BMI, body mass index; CHD, coronary heart disease; SEP, socio‐economic position; SD, standard deviation.

### Ten‐year drinking typologies

Regression coefficients of the associations between 10‐year alcohol typologies and trajectories of CRP, IL‐6 and IL‐1 RA are shown in Table 2.

**Table 2:** Fixed‐effect coefficients from linear mixed model regression for association between 10‐year drinking typologies and inflammatory marker trajectories during the following 12 years

	Log_e_ C‐reactive protein (mg L^−1^)		Log_e_ interleukin‐6 (ng mL^−1^)		Log_e_ interleukin‐1 receptor antagonist (ng mL^−1^)	
	Age and sex adjusted	Multivariable adjusted	Age and sex adjusted	Multivariable^a^adjusted	Age and sex adjusted	Multivariable adjusted
Intercept	−0.280*** (−0.316, −0.244)	−0.679*** (−0.725, −0.632)	0.329*** (0.310, 0.348)	0.147*** (0.122, 0.173)	5.554*** (5.534, 5.573)	5.408*** (5.381, 5.435)
Stable nondrinker	0.231*** (0.142, 0.320)	0.146** (0.057, 0.236)	0.174*** (0.124, 0.224)	0.099*** (0.048, 0.151)	0.072* (0.010, 0.134)	0.065* (0.007, 0.123)
Stable moderate drinker	0.000 (ref)	0.000 (ref)	0.000 (ref)	0.000 (ref)	0.000 (ref)	0.000 (ref)
Stable heavy drinker	0.251*** (0.160, 0.343)	0.156*** (0.067, 0.244)	0.142*** (0.094, 0.191)	0.109*** (0.060, 0.157)	0.033 (−0.016, 0.083)	−0.005 (−0.053, 0.044)
Nonstable drinker	0.133*** (0.074, 0.192)	0.075* (0.018, 0.132)	0.064*** (0.033, 0.095)	0.039* (0.008, 0.070)	0.011 (−0.022, 0.044)	−0.009 (−0.040, 0.023)
Former drinker	0.121** (0.035, 0.207)	0.071 (−0.011, 0.154)	0.118*** (0.073, 0.164)	0.089*** (0.044, 0.135)	0.045 (−0.007, 0.097)	0.041 (−0.009, 0.092)
Time (per year)	0.033*** (0.030, 0.037)	0.027*** (0.024, 0.031)	0.020*** (0.018, 0.022)	0.019*** (0.017, 0.021)	0.034*** (0.032, 0.036)	0.031*** (0.028, 0.033)
Stable nondrinker × time	0.004 (−0.005, 0.012)	0.004 (−0.005, 0.013)	−0.002 (−0.007, 0.004)	−0.000 (−0.006, 0.005)	0.002 (−0.005, 0.009)	0.004 (−0.002, 0.011)
Stable moderate drinker × time	0.000 (ref)	0.000 (ref)	0.000 (ref)	0.000 (ref)	0.000 (ref)	0.000 (ref)
Stable heavy drinker × time	−0.005 (−0.013, 0.004)	−0.005 (−0.014, 0.004)	0.010*** (0.005, 0.016)	0.009** (0.003, 0.014)	0.004 (−0.002, 0.010)	0.005 (−0.001, 0.011)
Nonstable drinker × time	−0.003 (−0.008, 0.003)	−0.003 (−0.008, 0.003)	−0.004* (−0.007, −0.000)	−0.004* (−0.007, −0.000)	0.000 (−0.004, 0.004)	0.001 (−0.003, 0.005)
Former drinker × time	0.003 (−0.006, 0.011)	0.003 (−0.006, 0.011)	−0.004 (−0.009, 0.001)	−0.004 (−0.009, 0.001)	0.005 (−0.000, 0.011)	0.005 (−0.001, 0.010)
*N* (no. of observations)	8128 (19 097)	7814 (18 097)	8183 (19 182)	7868 (18 179)	3468 (9564)	3443 (9244)

***P < 0.001; **P < 0.01; *P < 0.05.

a Multivariable adjustment for age, sex, ethnicity, prevalent coronary heart disease or type 2 diabetes at phase 3, socio‐economic position, smoking status, physical activity, diet and body mass index.

In multivariable‐adjusted models, all drinking typologies other than former drinkers had statistically significantly elevated levels of CRP at phase 3 in comparison with stable moderate drinkers. No drinking groups differed significantly in their rate of change in CRP over time.

All drinking typologies had elevated levels of IL‐6 at phase 3 compared to moderate drinkers in the multivariable‐adjusted model (10.4% [95% confidence interval (95% CI), 4.9–16.3] higher in stable nondrinkers, 11.5% [95% CI, 6.2–17.0] higher in stable heavy drinkers and 9.3% [95% CI, 4.5–14.5] higher in former drinkers). Stable heavy drinkers also showed increases in the rate of change in IL‐6 levels over time [0.9% (95% CI 0.3–1.4) per year]. Nonstable drinkers had slightly shallower increases in their rate of change in IL‐6 over time [−0.4% (95% CI, 0.7 to ~0.0)] compared to moderate drinkers.

Stable nondrinkers had elevated levels of IL‐1 RA compared to moderate drinkers at phase 3 [6.7% (95% CI, 0.7–13.1)]. No significant effects on the levels of IL‐1 RA were observed for any other drinking typology, nor were any significant effects observed for any drinking typology on the rate of change in IL‐1 RA over time.

**Figure 1:**
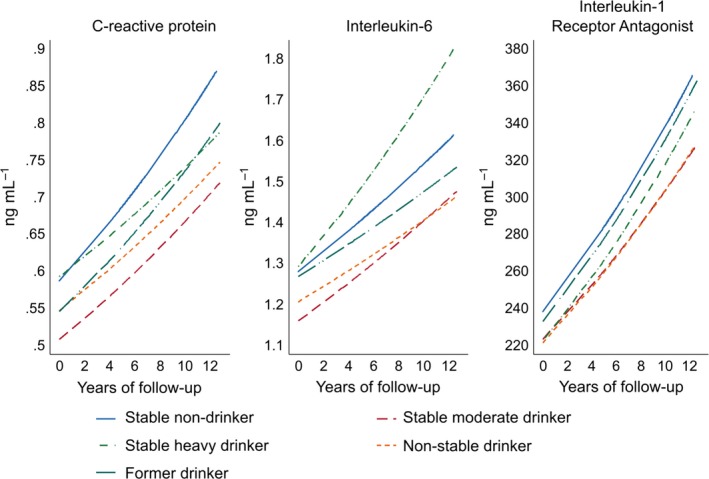
Model‐predicted C‐reactive protein, interleukin‐6 and interleukin‐1 receptor antagonist trajectories by 10‐year drinking typologies. Multivariable adjustment for age, sex, ethnicity, prevalent coronary heart disease or type 2 diabetes at phase 3, socio‐economic position, smoking status, physical activity, diet and body mass index. Graphs are based on the back transformation of log‐transformed C‐reactive protein, interleukin‐6 and interleukin‐1 receptor antagonist predicted by linear mixed models (Table 2).

The mean trajectories of each biomarker, predicted using the multivariable model, by drinking typology are presented graphically in Fig. 1.

Findings based on drinking information alone at study phase 3 were similar to those observed using 10‐year drinking typologies (Table S2 and Figure S1). However, whilst the findings were broadly concordant, those based on information on drinking behaviour over a 10‐year period revealed that participants with a sustained history of heavy drinking had higher levels of inflammation as well as steeper increases in IL‐6 values than were seen when using current drinking information alone. As a *post hoc* analysis suggested by reviewers for further comparison with evidence from small‐scale feeding trials 28, we repeated the analysis for changes in fibrinogen during an average period of ~5 years. We found that compared to moderate drinkers, both former drinkers and stable nondrinkers had higher levels throughout follow‐up. Conversely, heavy drinkers had, on average, the lowest levels of fibrinogen during observation (Tables S3, S4 and Figure S2).

## Discussion

### Summary of findings

We observed that those who consistently consumed alcohol at levels considered moderate over a 10‐year period had lower concentrations of CRP, IL‐6 and IL‐1 RA compared to nondrinkers during the following 12 years. Former drinkers also had higher levels of each of these markers during this period, although this association was not statistically significant. Additionally, we found that heavy drinkers not only had increased levels of these cytokines compared to stable moderate drinkers, but also demonstrated a more marked rate of change in IL‐6 levels over time.

### Interpretation, comparison to other work and implications

Our findings are broadly consistent with the U‐ or J‐shaped associations observed between alcohol consumption and cardiometabolic outcomes. Thus, we found that nondrinkers, former drinkers and heavy drinkers had consistently elevated levels of CRP, IL‐6 and IL‐1 RA over a 12‐year period. These findings are supported by evidence from other studies of the effect of alcohol consumption on inflammatory markers 30, 31. Two of these markers (IL‐6 and IL‐1 RA) are believed to be causally associated with CHD, providing indirect evidence that moderate alcohol consumption may confer some cardioprotective effects. The mechanisms by which alcohol achieves this are unknown, but may involve mild activation of inflammatory pathways, which confer some benefit. On the other hand, these pathways are also likely to be involved in the detrimental effects of heavy drinking. These observations are concordant with large‐scale Mendelian randomization studies that have shown that inhibiting IL‐6 signalling could reduce the risk of developing CHD 53 whilst inhibiting IL‐1 α/β may increase the risk of CHD 26. This highlights not only the complexity of inflammatory pathways underlying CVDs but also the complicated role of alcohol consumption in the development of such diseases. For example, moderate alcohol intake may induce oxidative stress by induction of particular heat shock proteins, which inhibit the activation of various pro‐inflammatory cytokines leading to endotoxin tolerance 54. Endotoxin tolerance is thought to be a protective mechanism against developing coronary occlusion and acute coronary syndromes 55, and may explain why moderate drinkers have a lower risk of CHD than nondrinkers. By contrast, a pattern of heavy alcohol use may lead to low‐level gut bacterial translocation and consequent increased levels of pro‐inflammatory cytokines. It was recently demonstrated that an acute alcohol ‘binge’ of 0.8 g kg^−1^ in healthy individuals led to a rapid increase in serum endotoxin and bacterial DNA, as well as increased acute‐phase protein levels and pro‐inflammatory cytokine responses 56. This pattern of low‐level bacterial translocation has been associated with disease progression in animal models of obesity and metabolic syndrome 57, 58.

In terms of overall effect sizes, the multivariable‐adjusted effect of stable nondrinking on the intercept of IL‐6 is equivalent to the per‐allele effect of single nucleotide polymorphism (SNP) rs7529229 in the *IL6R* gene (Ch1q21.3) on IL‐6 levels 53 which confers an approximately 5% reduction in odds of developing CHD. CRP levels were increased by 15.7% in nondrinkers compared to stable moderate drinkers, which is similar to the per‐allele difference observed for *CRP* SNP rs1130864 59. However, this elevated level of CRP has been shown not to confer a protective or detrimental effect for CHD 59. The effect of nondrinking on IL‐1 RA although statistically significant was relatively small, and less than the effect observed for a genetic allele count score using two common, uncorrelated, SNPs (rs6743376 and rs1542176) located upstream of *ILRN* (the gene that encodes the IL‐1 RA) 26. Given that a per‐allele increase in this genetic score was only associated with a 3% increase in the odds of developing CHD and not significantly associated with developing type 2 diabetes 26, the relative contribution of moderate drinking to a reduced risk of CHD via IL‐1 α/β is likely to be minimal.

Our longitudinal approach demonstrates the importance of accounting for repeat measures of alcohol intake when estimating associations with health outcomes 35. We found that only considering alcohol consumption at one point in time led to underestimation of the effect of heavy drinking on inflammatory markers. Furthermore, we observed that former drinkers had higher levels of cytokines than stable nondrinkers, in keeping with the idea that failing to exclude former drinkers from the group of stable nondrinkers will lead to overestimating the protective effect of moderate drinking 42. This group may consist of former heavy drinkers (including individuals with a history of alcohol use disorders) and those with illnesses that have led to alcohol cessation 60, both of which are likely to be associated with higher levels of inflammation 61, 62, 63. However, it is also worth noting that amongst those with existing illness, alcohol abstinence is generally associated with better long‐term prognosis than continued drinking 64, 65. Furthermore, whilst it has been shown that CVD risk factors are more common in nondrinkers 66, even when lifelong nondrinkers can be separated from former and occasional drinkers, ill‐health prior to the age at which alcohol consumption typically begins has been shown to be more common in those who have never consumed alcohol 67. All of these factors may contribute to higher levels of inflammation as well as increased risk of CVD in nondrinkers. As such, the protective effect of moderate drinking on cardiometabolic outcomes is likely to be more modest than is often believed 41, 68.

### Strengths and weaknesses

Our study has several strengths including the large sample size and use of repeat measures of alcohol consumption to define alcohol typologies over a 10‐year period (reducing drinking category misclassification bias inherent in studies utilizing only one measure of alcohol consumption) as well as repeat measures of markers of inflammation.

However, there are also several shortcomings. For example, it is known that heavy drinkers are underrepresented in population‐level surveys; the drinkers in our sample are typically low‐to‐moderate consumers 35. This means that the effect we observed for heavy drinking on trajectories of inflammatory markers is likely to be an underestimate of the ‘true’ association.

Another limitation is that we used self‐reported measures of alcohol consumption which have been criticized 69. Furthermore, we focused on total weekly quantity of alcohol intake, not changes in both frequency and quantity. It is plausible that shifts in pattern of consumption may better explain differences in risk of CHD over time 70. However, this does not affect our comparison with existing work which has also mostly focused on singular components of drinking behaviour (also largely overall weekly quantity of alcohol consumption).

Finally, participants in the Whitehall II study are not a representative sample of the general population; however, it has been shown that cardiometabolic‐related aetiological findings from this cohort are broadly consistent with those obtained from representative cohorts 71.

## Conclusion

Stable moderate alcohol consumption is associated with a shift in the long‐term inflammatory marker profile which is consistent with conferring a reduced risk of developing CHD. The implication of this is that modulation of chronic inflammation is a significant pathway through which alcohol affects overall CHD risk.

## Conflict of interest statement

No conflicts of interest to declare.

## Sources of funding

This work was supported by grants from the European Research Council (ERC‐StG‐2012‐309337_AlcoholLifecourse, PI: Annie Britton, http://www.ucl.ac.uk/alcohol-lifecourse) and UK Medical Research Council/Alcohol Research UK (MR/M006638/1). The Whitehall II study was supported by grants from the UK Medical Research Council (K013351), British Heart Foundation (RG/07/008/23674), Stroke Association, National Heart Lung and Blood Institute (HL036310) and National Institute on Aging (AG13196, AG034454). The funders had no role in the design of the study, data collection and analysis, or the preparation of and decision to publish the manuscript.

## Author contributions

SB and AB conceived the research question. SB carried out the analysis. SB completed the first draft of the manuscript. AB, GM and KM provided additional intellectual content. All authors read and agreed on the final submitted manuscript.

## Supporting information


**Table S1.** Descriptive information of changes in inflammatory markers and alcohol consumption during follow‐up by drinking typology.
**Table S2.** Fixed‐effect coefficients from linear mixed model regression for association of current drinking habits and inflammatory marker trajectories during the following 12 years.
**Table S3.** Fixed‐effect coefficients from linear mixed model regression for association of current drinking category and fibrinogen trajectories during the following 7 years.
**Table S4.** Fixed‐effect coefficients from linear mixed model regression for association of ten year drinking typologies and fibrinogen trajectories during the following 7 years.
**Figure S1.** Model predicted C‐reactive protein, interleukin‐6 and interleukin‐1 receptor antagonist trajectories by current drinking status.
**Figure S2.** Multivariable adjusted model predicted fibrinogen trajectories by current drinking status (left) and ten year drinking typology (right).
