# Supplementary material for: Ten‐year alcohol consumption typologies and trajectories of C‐reactive protein, interleukin‐6 and interleukin‐1 receptor antagonist over the following 12 years: a prospective cohort study
Source: J Intern Med. 2016 Aug 3;281(1):75–85. doi: 10.1111/joim.12544 (PMC5173424; doi:10.1111/joim.12544)
Supplement: Supplementary file 1 — Table S1. Descriptive information of changes in inflammatory markers and alcohol consumption during follow‐up by drinking typology. Table S2. Fixed‐effect coefficients from linear mixed model regression for association of current drinking habits and inflammatory marker trajectories during the following 12 years. Table S3. Fixed‐effect coefficients from linear mixed model regression for association of current drinking category and fibrinogen trajectories during the following 7 years. Table S4. Fixed‐effect coefficients from linear mixed model regression for association of ten year drinking typologies and fibrinogen trajectories during the following 7 years. Figure S1. Model predicted C‐reactive protein, interleukin‐6 and interleukin‐1 receptor antagonist trajectories by current drinking status. Figure S2. Multivariable adjusted model predicted fibrinogen trajectories by current drinking status (left) and ten year drinking typology (right). [file JOIM-281-75-s001.docx]

*Journal of Internal Medicine – Original article*

Supplementary material for: **Ten year alcohol consumption typologies and trajectories of C-reactive protein, interleukin-6, and interleukin-1 receptor antagonist over the following twelve years: a prospective cohort study**

*Authors and affiliations*

Steven Bell^1^, Gautam Mehta^2^, Kevin Moore^2^, Annie Britton^1^

^1^ Research Department of Epidemiology and Public Health, University College London, 1-19 Torrington Place, London, WC1E6BT, United Kingdom.

^2^ UCL Institute of Liver and Digestive Health, Royal Free Campus, University

College London, Rowland Hill Street, London, NW3 2PF, United Kingdom.

**Keywords:** alcohol, cytokines, inflammation, longitudinal, epidemiology

eTable 1 - Descriptive information of changes in inflammatory markers and alcohol consumption during follow-up by drinking typology

|  | Stable non-drinker | | Stable moderate drinker | | Stable heavy drinker | | Non-stable drinker | | Former drinker | | Total | |
| --- | --- | --- | --- | --- | --- | --- | --- | --- | --- | --- | --- | --- |
|  | N | Mean (SD) | N | Mean (SD) | N | Mean (SD) | N | Mean (SD) | N | Mean (SD) | N | Mean (SD) |
| **Alcohol (g) consumption (median, (25^th^, 75^th^ percentile)** |  |  |  |  |  |  |  |  |  |  |  |  |
| Phase 3 | 723 | 0 (0, 0) | 3832 | 48 (32, 48) | 660 | 272 (208, 272) | 2003 | 96 (24, 96) | 831 | 0 (0, 0) | 8049 | 48 (16, 48) |
| Phase 5 | 549 | 0 (0, 0) | 3285 | 80 (40, 80) | 557 | 288 (208, 288) | 1688 | 112 (32, 112) | 669 | 16 (0, 16) | 6748 | 72 (24, 72) |
| Phase 7 | 496 | 0 (0, 0) | 3180 | 72 (32, 72) | 522 | 240 (160, 240) | 1623 | 96 (32, 96) | 611 | 16 (0, 16) | 6432 | 64 (16, 64) |
| **log_e_ C-reactive protein (mg/l)** |  |  |  |  |  |  |  |  |  |  |  |  |
| Phase 3 | 669 | 0.05 (1.12) | 3,534 | -0.24 (1.12) | 608 | -0.02 (1.11) | 1,841 | -0.12 (1.09) | 728 | -0.09 (1.14) | 7,380 | -0.15 (1.11) |
| Phase 5 | 466 | 0.21 (1.08) | 2,928 | -0.10 (1.05) | 492 | 0.02 (1.11) | 1,487 | -0.01 (1.03) | 567 | 0.05 (1.09) | 5,940 | -0.03 (1.06) |
| Phase 7 | 436 | 0.44 (0.96) | 2,876 | 0.11 (0.99) | 468 | 0.29 (0.99) | 1,459 | 0.19 (0.96) | 538 | 0.29 (1.01) | 5,777 | 0.19 (0.99) |
| **log_e_ IL-6 (ng/l)** |  |  |  |  |  |  |  |  |  |  |  |  |
| Phase 3 | 680 | 0.56 (0.65) | 3,562 | 0.37 (0.60) | 613 | 0.47 (0.60) | 1,870 | 0.43 (0.60) | 760 | 0.51 (0.63) | 7,485 | 0.43 (0.61) |
| Phase 5 | 494 | 0.58 (0.64) | 3,029 | 0.37 (0.58) | 510 | 0.55 (0.63) | 1,531 | 0.38 (0.57) | 591 | 0.49 (0.62) | 6,155 | 0.41 (0.60) |
| Phase 7 | 409 | 0.77 (0.61) | 2,772 | 0.6 (0.58) | 467 | 0.82 (0.68) | 1,385 | 0.62 (0.59) | 509 | 0.68 (0.62) | 5,542 | 0.64 (0.60) |
| **log_e_ IL-1 RA (ng/l)** |  |  |  |  |  |  |  |  |  |  |  |  |
| Phase 3 | 264 | 5.65 (0.49) | 1,700 | 5.54 (0.41) | 272 | 5.56 (0.37) | 887 | 5.56 (0.43) | 335 | 5.63 (0.48) | 3,458 | 5.57 (0.43) |
| Phase 5 | 229 | 5.95 (0.52) | 1,560 | 5.85 (0.39) | 255 | 5.9 (0.42) | 808 | 5.86 (0.41) | 301 | 5.92 (0.43) | 3,153 | 5.87 (0.41) |
| Phase 7 | 213 | 6.03 (0.61) | 1,476 | 5.9 (0.49) | 228 | 5.96 (0.52) | 759 | 5.92 (0.58) | 277 | 6.04 (0.62) | 2,953 | 5.93 (0.54) |

IL-6 = Interleukin-6, IL-1 RA = Interleukin-1 receptor antagonist

**eTable 2 - Fixed-effect coefficients from linear mixed model regression for association of current drinking habits and inflammatory marker trajectories during the following 12 years**

|  | log_e_ C-reactive protein (mg/l) | | log_e_ Interleukin-6 (ng/ml) | | log_e_ Interleukin-1 Receptor Antagonist (ng/ml) | |
| --- | --- | --- | --- | --- | --- | --- |
|  | Age and sex adjusted | Multivariable adjusted | Age and sex adjusted | Multivariable adjusted | Age and sex adjusted | Multivariable adjusted |
| **Intercept** | -0.261*** [-0.293,-0.229] | -0.671*** [-0.715,-0.627] | 0.345*** [0.328,0.362] | 0.157*** [0.133,0.181] | 5.558*** [5.541,5.575] | 5.409*** [5.383,5.435] |
| Non-drinker | 0.191*** [0.103,0.279] | 0.133** [0.045,0.220] | 0.154*** [0.104,0.203] | 0.090*** [0.039,0.140] | 0.068* [0.007,0.129] | 0.066* [0.008,0.123] |
| Former drinker | 0.099* [0.016,0.183] | 0.059 [-0.022,0.139] | 0.103*** [0.059,0.148] | 0.079*** [0.034,0.123] | 0.041 [-0.010,0.092] | 0.041 [-0.008,0.090] |
| Moderate drinker | 0.000 [ref] | 0.000 [ref] | 0.000 [ref] | 0.000 [ref] | 0.000 [ref] | 0.000 [ref] |
| Heavy drinker | 0.207*** [0.140,0.275] | 0.143*** [0.078,0.207] | 0.084*** [0.048,0.120] | 0.067*** [0.032,0.103] | 0.009 [-0.029,0.048] | -0.023 [-0.060,0.014] |
| **Time (per year)** | 0.033*** [0.030,0.036] | 0.027*** [0.024,0.030] | 0.019*** [0.017,0.021] | 0.018*** [0.016,0.020] | 0.034*** [0.032,0.036] | 0.031*** [0.029,0.033] |
| Non-drinker × Time | 0.004 [-0.004,0.012] | 0.005 [-0.004,0.013] | -0.001 [-0.006,0.005] | 0.001 [-0.005,0.007] | 0.002 [-0.005,0.009] | 0.004 [-0.002,0.011] |
| Former drinker × Time | 0.003 [-0.005,0.011] | 0.003 [-0.005,0.011] | -0.002 [-0.007,0.003] | -0.003 [-0.008,0.003] | 0.005 [-0.000,0.011] | 0.004 [-0.001,0.010] |
| Moderate drinker × Time | 0.000 [ref] | 0.000 [ref] | 0.000 [ref] | 0.000 [ref] | 0.000 [ref] | 0.000 [ref] |
| Heavy drinker × Time | -0.006 [-0.012,0.001] | -0.005 [-0.012,0.001] | 0.007** [0.002,0.011] | 0.005* [0.001,0.010] | 0.002 [-0.003,0.006] | 0.003 [-0.002,0.007] |
| N (# obs) | 7,973 (18,855) | 7,806 (18,082) | 8,025 (18,936) | 7,860 (18,162) | 3,464 (9,556) | 3,442 (9,241) |

*** p < 0.001; ** p < 0.01; * p < 0.05

^†^ Multivariable = age, sex, ethnicity, prevalent CHD or type 2 diabetes at phase 3, socioeconomic position, smoking status, physical activity, diet and body mass index.


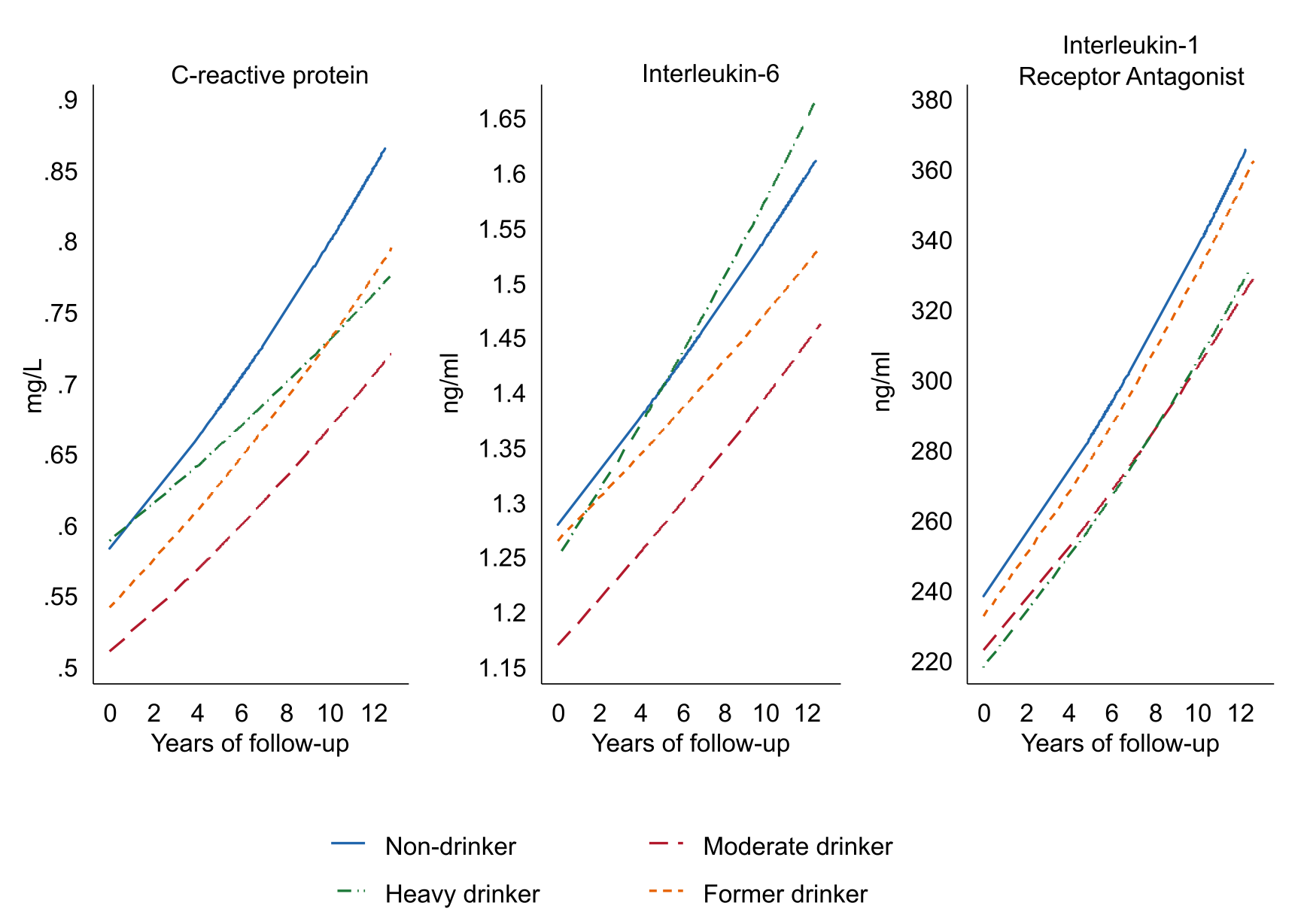


**eFigure 1 – Model predicted C-reactive protein, interleukin-6 and interleukin-1 receptor antagonist trajectories by current drinking status**

Multivariable = age, sex, ethnicity (white or non-white), prevalent CHD or type 2 diabetes at phase 3, socioeconomic position, smoking status, physical activity, diet and body mass index.

**Post hoc analyses of changes in fibrinogen**

Fibrinogen was assayed using a modification of the clotting method of Clauss. However, using a 1:15 dilution of plasma to 0.9% saline, clotted with a half volume of bovine thrombin, 50 U/mL, in an MDA-180 coagulator (OrganonTeknika, Cambridge, UK) using the manufacturer’s reagents and the international fibrinogen standard. Fibrinogen was measured at study phases 3 (1991-1994) and 5 (1997-1999). We used natural log transformed values for all analyses.

Models were estimated as described in the main manuscript (with the exception that we did not allow for a random effect of time given fibrinogen was only assessed at 2 occasions).

**Current drinking only**

**eTable 3 - Fixed-effect coefficients from linear mixed model regression for association of current drinking category and fibrinogen trajectories during the following 7 years.**

|  | log_e_ Fibrinogen (g/l) |  |
| --- | --- | --- |
|  | Age and sex adjusted | Multivariable^†^ adjusted |
| **Intercept** | 0.828 [0.821,0.835]*** | 0.784 [0.775,0.794]*** |
| Non-drinker | 0.067 [0.049,0.085]*** | 0.056 [0.037,0.075]*** |
| Former drinker | 0.045 [0.027,0.062]*** | 0.041 [0.023,0.058]*** |
| Moderate drinker | 0.000 [ref] | 0.000 [ref] |
| Heavy drinker | -0.024 [-0.038,-0.010]*** | -0.031 [-0.045,-0.017]*** |
| **Time (per year)** | 0.042 [0.041,0.043]*** | 0.041 [0.040,0.042]*** |
| Non-drinker × Time | -0.001 [-0.004,0.003] | -0.000 [-0.004,0.003] |
| Former drinker × Time | -0.003 [-0.006,0.000] | -0.003 [-0.007,0.000] |
| Moderate drinker × Time | 0.000 [ref] | 0.000 [ref] |
| Heavy drinker × Time | 0.000 [-0.002,0.003] | 0.000 [-0.002,0.003] |
| N (# observations) | 7,918 (13,316) | 7,672 (12,645) |

*** p < 0.001; ** p < 0.01; * p < 0.05

^†^ Multivariable = age, sex, ethnicity (white or non-white), prevalent CHD or type 2 diabetes at phase 3, socioeconomic position, smoking status, physical activity, diet and body mass index.

**Drinking typologies**

**eTable 4 - Fixed-effect coefficients from linear mixed model regression for association of ten year drinking typologies and fibrinogen trajectories during the following 7 years.**

|  | log_e_ Fibrinogen (g/l) |  |
| --- | --- | --- |
|  | Age and sex adjusted | Multivariable^†^ adjusted |
| **Intercept** | 0.823 [0.816,0.831]*** | 0.780 [0.770,0.790]*** |
| Stable non-drinker | 0.072 [0.054,0.090]*** | 0.060 [0.040,0.079]*** |
| Stable moderate drinker | 0.000 [ref] | 0.000 [ref] |
| Stable heavy drinker | -0.021 [-0.040,-0.002]* | -0.030 [-0.049,-0.010]** |
| Non-stable drinker | 0.011 [-0.001,0.023] | 0.005 [-0.007,0.018] |
| Former drinker | 0.049 [0.031,0.066]*** | 0.045 [0.027,0.063]*** |
| **Time (per year)** | 0.042 [0.041,0.043]*** | 0.042 [0.040,0.043]*** |
| Stable non-drinker × Time | -0.001 [-0.005,0.002] | -0.001 [-0.004,0.003] |
| Stable moderate drinker × Time | 0.000 [ref] | 0.000 [ref] |
| Stable heavy drinker × Time | -0.000 [-0.003,0.003] | -0.000 [-0.004,0.003] |
| Non-stable drinker × Time | -0.002 [-0.004,0.001] | -0.001 [-0.004,0.001] |
| Former drinker × Time | -0.003 [-0.007,-0.000]* | -0.004 [-0.007,-0.000]* |
| N (# observations) | 8,044 (13,468) | 7,679 (12,655) |

*** p < 0.001; ** p < 0.01; * p < 0.05
 ^†^ Multivariable = age, sex, ethnicity (white or non-white), prevalent CHD or type 2 diabetes at phase 3, socioeconomic position, smoking status, physical activity, diet and body mass index.


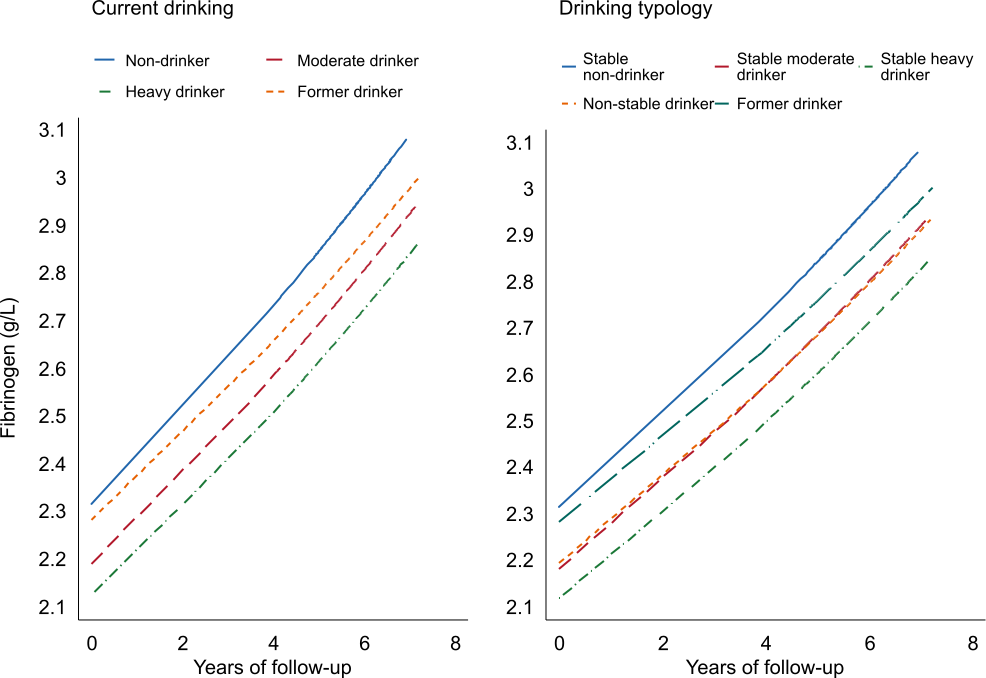


**eFigure 2 – Multivariable adjusted model predicted fibrinogen trajectories by current drinking status (left) and ten year drinking typology (right)**
